# Fundus autofluorescence of retinal angiomatous proliferation

**DOI:** 10.1371/journal.pone.0243458

**Published:** 2020-12-09

**Authors:** Masaaki Saito, Kanako Itagaki, Tetsuju Sekiryu

**Affiliations:** 1 Department of Ophthalmology, Fukushima Medical University School of Medicine, Fukushima, Japan; 2 Department of Ophthalmology, Hirosaki University Graduate School of Medicine, Hirosaki, Japan; University of California Los Angeles, UNITED STATES

## Abstract

**Purpose:**

The present study aimed to evaluate the characteristics of fundus autofluorescence in Japanese patients with retinal angiomatous proliferation (RAP).

**Methods:**

We retrospectively reviewed 100 eyes from 76 patients (male, n = 45; female, n = 31; age range, 50–94 years; mean ± standard deviation, 81.4 ± 6.4 years) with treatment-naïve RAP, which was diagnosed based on the identification of retinal–retinal anastomosis on early-phase fluorescein angiography or indocyanine green angiography (ICGA) and the identification of a hot spot on late-phase ICGA. RAP was classified into the following three stages: stage 1, proliferation of intraretinal capillaries originating from the deep retinal complex (intraretinal neovascularization); stage 2, growth of the retinal vessels into the subretinal space (subretinal neovascularization); and stage 3, clinically or angiographically observed choroidal neovascularization. In all cases, short-wavelength and near-infrared autofluorescence (SW-AF, NIR-AF) was evaluated using a confocal scanning laser ophthalmoscope.

**Results:**

The conditions of the 100 eyes were as follows: stage 1 RAP, n = 6 (6%); stage 2 RAP without retinal pigment epithelial detachment (PED), n = 40 (40%); stage 2 RAP with PED, n = 44 (44%); and stage 3 RAP, 10 (10%). On NIR-AF imaging, the number of abnormalities that were observed to correspond to the RAP lesions on ICGA (87 eyes, 87%) was significantly greater in comparison to SW-AF imaging (27 eyes, 27%). The mean follow-up period in all 76 patients was 39.2 months. In the 76 patients with unilateral disease, 21 (21%) eyes developed RAP in the fellow eye during the follow-up period. Among 18 eyes that were examined by both SW-AF and NIR-AF imaging before the onset of RAP lesions, NIR-AF imaging showed hypoautofluorescence in 15 (83%) eyes before the onset of RAP lesions.

**Conclusions:**

SW-AF and NIR-AF abnormalities may be related to the dysfunction of the photoreceptor/retinal pigment epithelium complex. Hypoautofluorescence on NIR-AF imaging may accurately indicate the presence or onset of RAP lesions.

## Introduction

Yannuzzi et al. [1], who first identified retinal angiomatous proliferation (RAP) in 2001, described the disease as a variant of exudative age-related macular degeneration (AMD). RAP has three differentiated stages that are characterized by clinical and angiographic features, and it is now considered that type 3 choroidal neovascularization (CNV) distinguishes it from the Gass CNV type 1 and 2 anatomic classifications [1–3]. Although the prevalence of RAP is low (i.e., 15% in Caucasian patients and 4.5% in Japanese patients [4]), the natural course of RAP is associated with poor visual outcomes in comparison to typical exudative AMD [5, 6].

Intravitreal ranibizumab (Lucentis, Genentech, Inc., San Francisco, CA, USA) or aflibercept (Eylea, Regeneron, Tarrytown, NY, USA, and Bayer, Berlin, Germany) are now administered worldwide, as evidence-based therapy for exudative AMD [7–9]. However, anti-vascular endothelial growth factor (VEGF) monotherapy using intravitreal ranibizumab or aflibercept for RAP requires repeated treatments. We hypothesized that the combination therapy of intravitreal anti-VEGF agents and photodynamic therapy may allow the visual acuity (VA) and retinal morphology of patients with RAP to be improved or maintained with fewer treatments [10–14]. However, retinal pigment epithelium (RPE) atrophy, which can result in reduced VA, has been reported to develop after treatment in patients with RAP [15–19]. In patients with unilateral RAP lesions, the fellow eye is at high risk of developing RAP lesions [20]. Thus, new examinations are needed to diagnose RAP before the onset of RAP lesions, and may be important for the management of patients with RAP.

Short-wavelength autofluorescence (SW-AF) is mainly derived from lipofuscin in the RPE, and is related to its functional and metabolic features [21, 22]. Piccolino et al. [23] wrote the first report on near-infrared fundus autofluorescence (NIR-AF), which was described as originating from the melanin in the RPE and choroid [24]. Although the characteristic changes on SW-AF or NIR-AF imaging have been reported in Stargardt’s disease [25], Best disease [26], central serous chorioretinopathy [27], AMD [28], and idiopathic CNV [29], the changes on SW-AF or NIR-AF imaging in patients with RAP have not been characterized.

The purpose of the current study was to evaluate the characteristics of SW-AF and NIR-AF imaging and investigate the early retinal changes before the onset of RAP lesions in Japanese patients with RAP using optical coherence tomography (OCT), and SW-AF and NIR-AF imaging.

## Methods

We retrospectively reviewed the fundus characteristics of 100 eyes in 76 patients (male, n = 45; female, n = 31; age range, 50–94 years; mean ± standard deviation, 81.4 ± 6.4 years) with treatment-naïve RAP at the Macula Services department of Fukushima Medical University Hospital. This study adhered to the tenets of the Declaration of Helsinki. The institutional ethics committees of Fukushima Medical University reviewed and approved this study.

All patients underwent a standardized examination, including color and red-free fundus photography, fluorescein angiography (FA), and indocyanine green angiography (ICGA), which was obtained using a fundus camera (TRC-50, Topcon, Tokyo, Japan) along with a confocal scanning laser ophthalmoscope (Heidelberg Retina Angiograph 2 [HRA 2], Heidelberg Engineering, Heidelberg, Germany) and OCT (Heidelberg Spectralis HRA+OCT, Heidelberg Engineering, Heidelberg, Germany). All patients provided their written informed consent after receiving a full explanation of the potential risks and benefits of FA and ICGA.

The clinical diagnosis of RAP was established based on the identification of retinal–retinal anastomosis on early-phase FA or ICGA and the identification of a hot spot on late-phase ICGA [1, 2]. We also classified the cases of RAP into three stages: stage 1, proliferation of intraretinal capillaries originating from the deep retinal complex (intraretinal neovascularization); stage 2, growth of retinal vessels into the subretinal space (subretinal neovascularization); and stage 3, clinically or angiographically observed CNV [1, 2]. Both eyes of patients with RAP were included. SW-AF and NIR-AF imaging were performed using an HRA 2. The excitation laser and detection filters were paired at 488 and 500 nm for SW-AF imaging and 787 and 800 nm for NIR-AF imaging. A series of 16 digital images with a field of 30 × 30 degrees (768 × 768 pixels) were averaged to obtain high-quality images. The presence of abnormalities was defined as the presence of hyperautofluorescence and/or hypoautofluorescence on SW-AF and NIR-AF imaging. To assess the co-localization between the RAP lesions on the ICGA images and the presence of abnormalities on SW-AF and NIR-AF imaging, we used a multimodal fundus imaging analysis that was obtained by Spectralis HRA + OCT and/or the Photoshop^®^ software program (version CS6, Adobe^®^, San Jose, CA, USA) to evaluate the vessels on fundus images. Two retina specialists (MS and KI) evaluated all SW-AF and NIR-AF images. When the specialists were not in agreement, a third reviewer (TS) evaluated the abnormalities. If the eyes had two or more RAP lesions, we evaluated the each lesion.

We used the best-corrected visual acuity (BCVA) measured with a Japanese standard decimal VA chart and calculated the mean BCVA using the logarithm of the minimum angle of resolution (logMAR) scale.

Fisher’s exact test was used to compare the sensitivity of SW-AF and NIR-AF imaging in the detection of abnormalities. P values of <0.05 were considered to indicate statistical significance.

## Results

Table 1 shows the baseline characteristics of the 76 study patients. The conditions of the eyes were classified as follows: stage 1 RAP, n = 6 (6%); stage 2 RAP without retinal pigment epithelial detachment (PED), n = 40 (40%); stage 2 RAP with PED, n = 44 (44%); and stage 3 RAP, n = 10 (10%). The numbers of RAP lesions were as follows: one, n = 93; two n = 6; three n = 1. The mean logMAR VA level at baseline was 0.62 ± 0.40 (range, 1.70–0.00). The mean greatest linear dimension measured by FA was 2,907 ± 1,646 μm. The raw data for the patient characteristics are presented in S1 Table.

**Table 1:** Baseline patient characteristics.

Characteristic	
No. patients	76
Women, no (%)	45 (59.2)
Men, no (%)	31 (40.8)
Age (years)	
Mean ± SD	81.4 ± 6.4
No. eyes	100
LogMAR BCVA	
Mean ± SD	0.62 ± 0.40
Median (interquartile range)	0.52 (0.30 to 1.00)
Range	1.70–0.00
Stage	
1, no (%)	6 (6)
2, no (%)	40 (40.0)
2 with PED, no (%)	44 (44.0)
3, no (%)	10 (10.0)
GLD (μm)	
Mean ± SD	2,907 ± 1,646
PED, no (%)	54 (54.0)

SD, standard deviation; logMAR BCVA, logarithm of the minimum angle of resolution best-corrected visual acuity; PED, pigment epithelial detachment; GLD, greatest linear dimension.

### SW-AF

**Fig 1:**
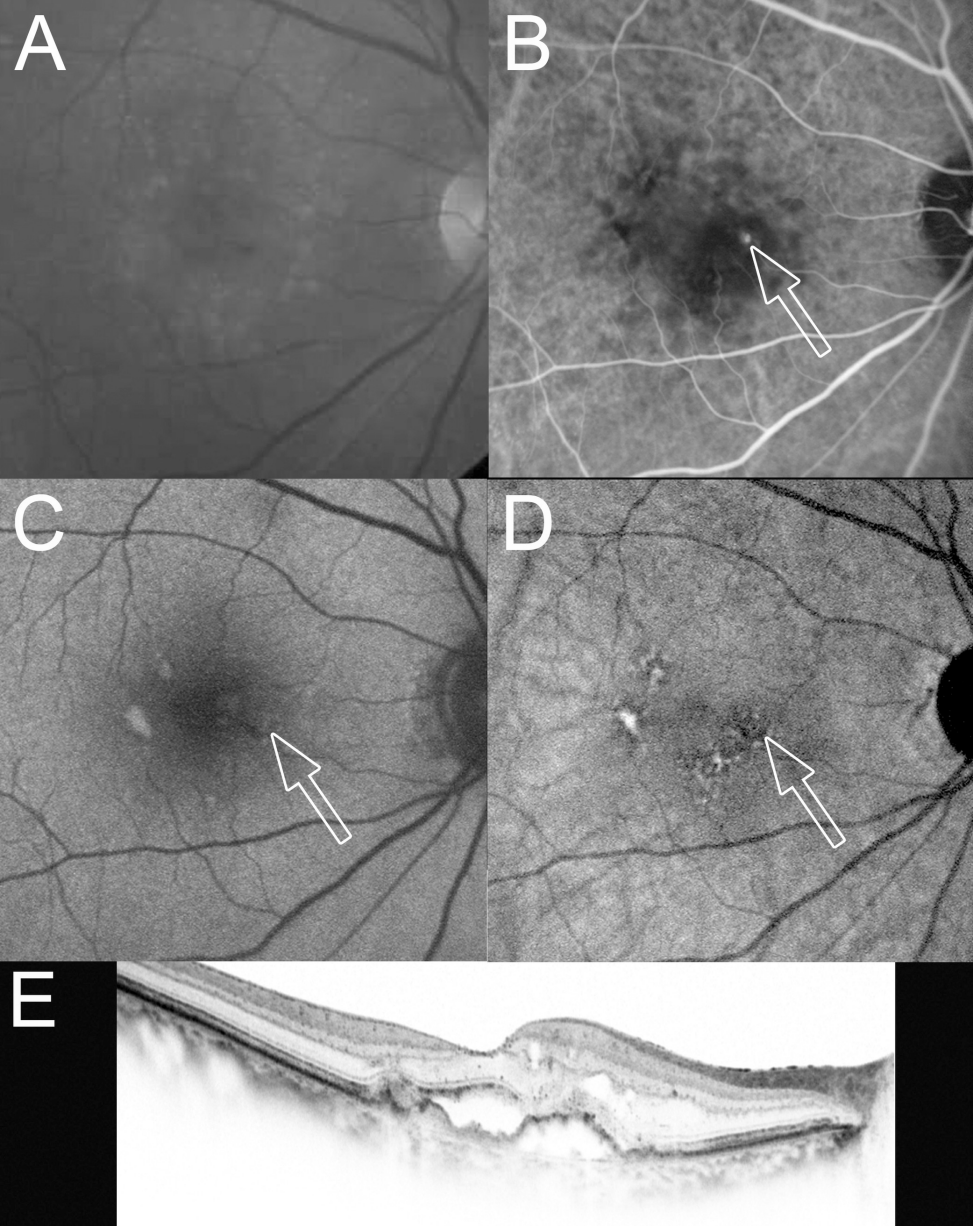
An 83-year-old woman with stage 2 RAP with PED at the time of the initial treatment. At the initial treatment, the BCVA (Snellen equivalent) was 1.0 decimal VA (20/20) in the right eye with stage 2 RAP with PED. (A) A red-free fundus photograph shows intraretinal hemorrhage, drusen, and PED in the macular area. (B) An early-phase ICGA image shows retinal–retinal anastomosis and an RAP lesion as a focal area of intense hyperfluorescence (hot spot, arrow). (C) An SW-AF image shows no abnormalities corresponding to the RAP lesion on ICGA (arrow). (D) An NIR-AF image shows hypoautofluorescence (arrow) corresponding to the RAP lesion on the ICGA image. (E) A horizontal OCT image shows cystoid macular edema, serous retinal detachment, and PED. Fluorescein angiography-guided photodynamic therapy was applied (laser spot size, 2,400 μm) 2 days after an intravitreal injection of ranibizumab followed by additional intravitreal injections of ranibizumab once per month for the next 2 months.

**Fig 2:**
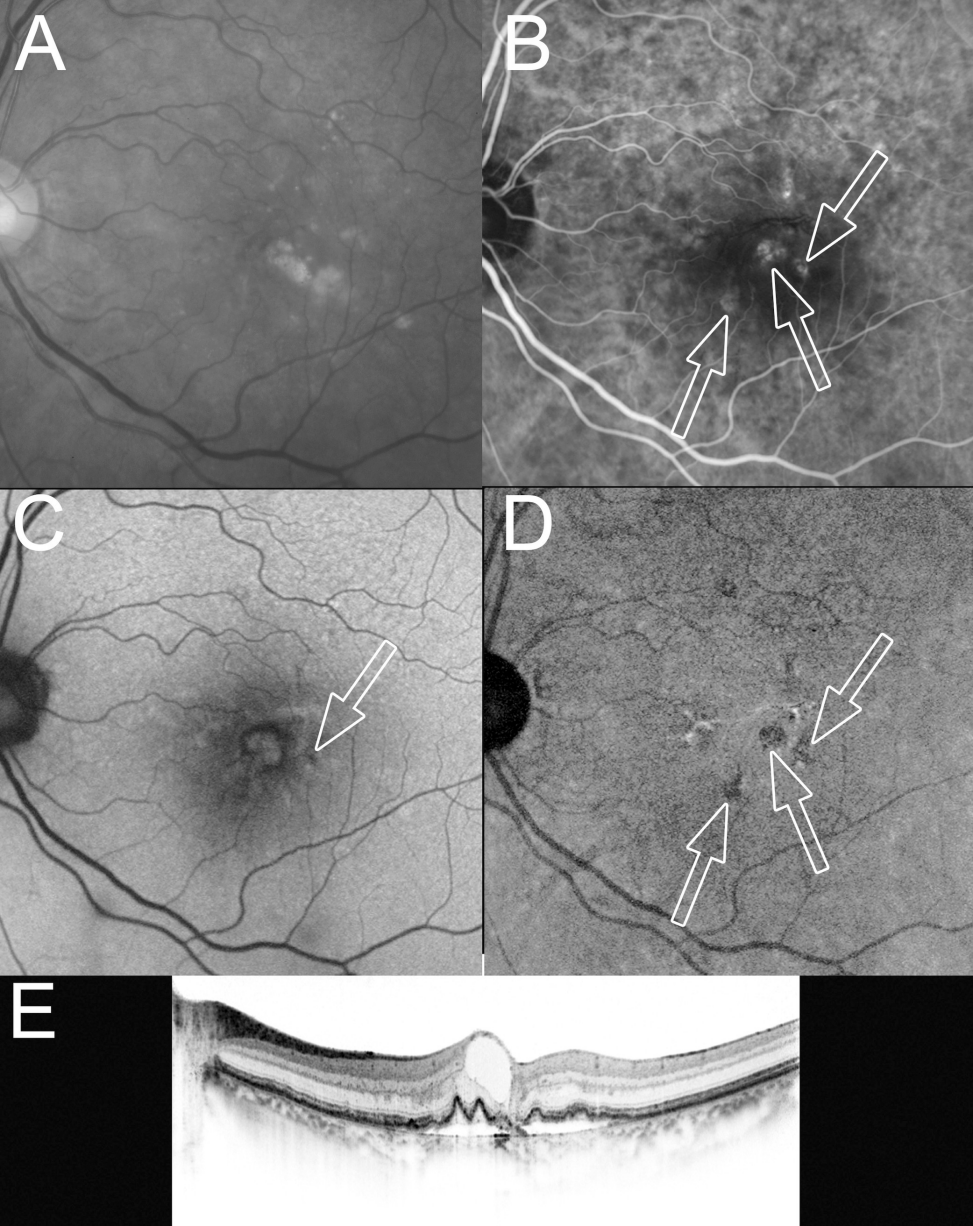
A 76-year-old woman with stage 2 RAP at the time of the initial treatment. At the initial treatment, the BCVA (Snellen equivalent) was 0.7 decimal VA (20/29) in the left eye with stage 2 RAP. (A) A red-free fundus photograph shows drusen at the macular area. (B) An early-phase ICGA image shows retinal-retinal anastomosis and RAP lesions as a focal area of intense hyperfluorescence (hot spots, arrows). (C) An SW-AF image shows a minute spot of hypoautofluorescence corresponding to one of the RAP lesions on the ICGA (arrow) image. (D) An NIR-AF image shows hypoautofluorescence (arrows) corresponding to all of the RAP lesions in the ICGA image. (E) A horizontal OCT image shows cystoid macular edema and elevation of the RPE line. Fluorescein angiography-guided photodynamic therapy was applied (laser spot size, 2,050 μm) 2 days after an intravitreal injection of ranibizumab, followed by additional intravitreal injections of ranibizumab that were administered once per month for the next 2 months.

Abnormalities corresponding to the RAP lesions on ICGA were seen on SW-AF imaging in 27 (27%) eyes (Table 2, Figs 1 and 2). The following abnormalities were observed: hyperautofluorescence, n = 1; hypoautofluorescence, n = 24; both hyperautofluorescence and hypoautofluorescence, n = 1; and hypoautofluorescence and blockage, n = 1 (Figs 1C and 2C). In 13 (13%) eyes, preretinal hemorrhage blocked the autofluorescence. SW-AF imaging showed no abnormalities in 60 (60%) eyes. The raw data for the SW-AF and NIR-AF imaging abnormalities are presented in S2 Table.

**Table 2:** Abnormalities corresponding to the RAP lesions on indocyanine green angiography (100 eyes).

	SW-AF	NIR-AF	P Value*
Abnormalities; eyes (%)	27 (27%)	87 (87%)	<0.0001
Hyperautofluorescence; eyes (%)	1 (1%)	1 (1%)	1.00
Hypoautofluorescence; eyes (%)	24 (24%)	82 (82%)	<0.0001
Hyperautofluorescence and	1 (1%)	2 (2%)	1.00
hypoautofluorescence; eyes (%)			
Hypoautofluorescence and block; eyes (%)	1 (1%)	2 (2%)	1.00
Blockage, eyes (%)	13 (13%)	4 (4%)	0.040
None, eyes (%)	60 (60%)	9 (9%)	<0.0001

*Fisher’s exact test.

RAP, retinal angiomatous proliferation; SW-AF, short-wavelength autofluorescence; NIR-AF, near-infrared fundus autofluorescence.

### NIR-AF

Abnormalities corresponding to the RAP lesions on ICGA were seen in 87 (87%) eyes on NIR-AF imaging (Table 2). The abnormalities of the 87 eyes were as follows: hyperautofluorescence, n = 1; hypoautofluorescence, n = 82; both hyperautofluorescence and hypoautofluorescence, n = 2; and hypoautofluorescence and blockage, n = 2 (Figs 1D and 2D). Preretinal hemorrhage blocked the hypoautofluorescence in 4 (4%) eyes. NIR-AF imaging showed no abnormalities in 9 (9%) eyes. Significant differences between SW-AF and NIR-AF imaging were seen with regard to the prevalence of abnormalities, the prevalence of hypoautofluorescence, the prevalence of blockage, and the prevalence of eyes with no abnormalities (Fisher’s exact test: p<0.0001, p<0.0001, p = 0.040, and p<0.0001, respectively) (Table 2).

### SW-AF and NIR-AF imaging findings before the onset of RAP lesions

**Fig 3:**
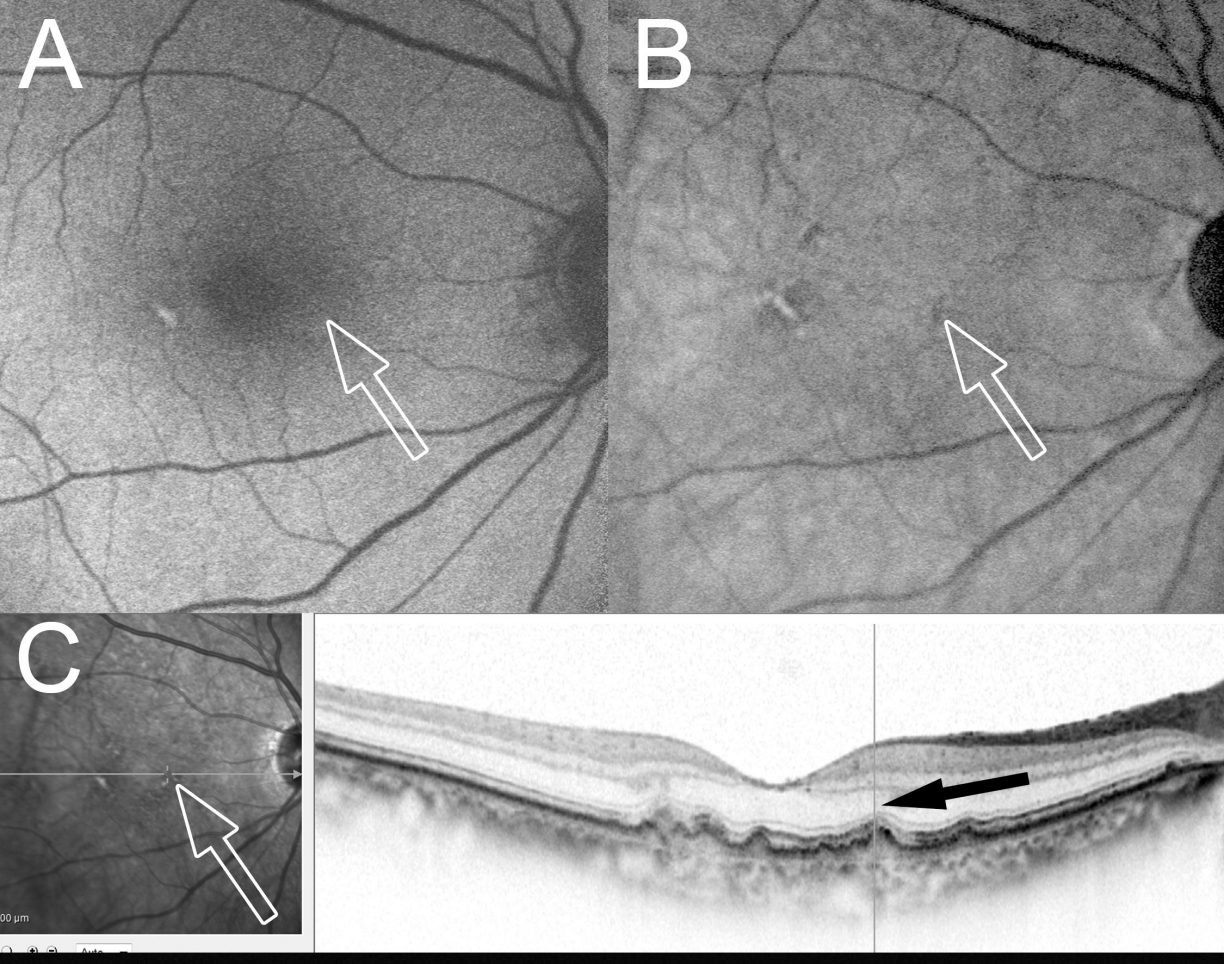
SW-AF and NIR-AF images obtained 37 months before the onset of RAP lesions. Thirty-seven months before the initial treatment in the case shown in Fig 1. The arrows in A, B, and C correspond to the RAP lesions on the baseline ICGA images. (A) An SW-AF image shows no abnormalities (arrow). (B) An NIR-AF image shows hypoautofluorescence (arrow). (C) A horizontal OCT image shows bulging of the RPE line and thinning of the outer nuclear layer (ONL) (black arrow).

**Fig 4:**
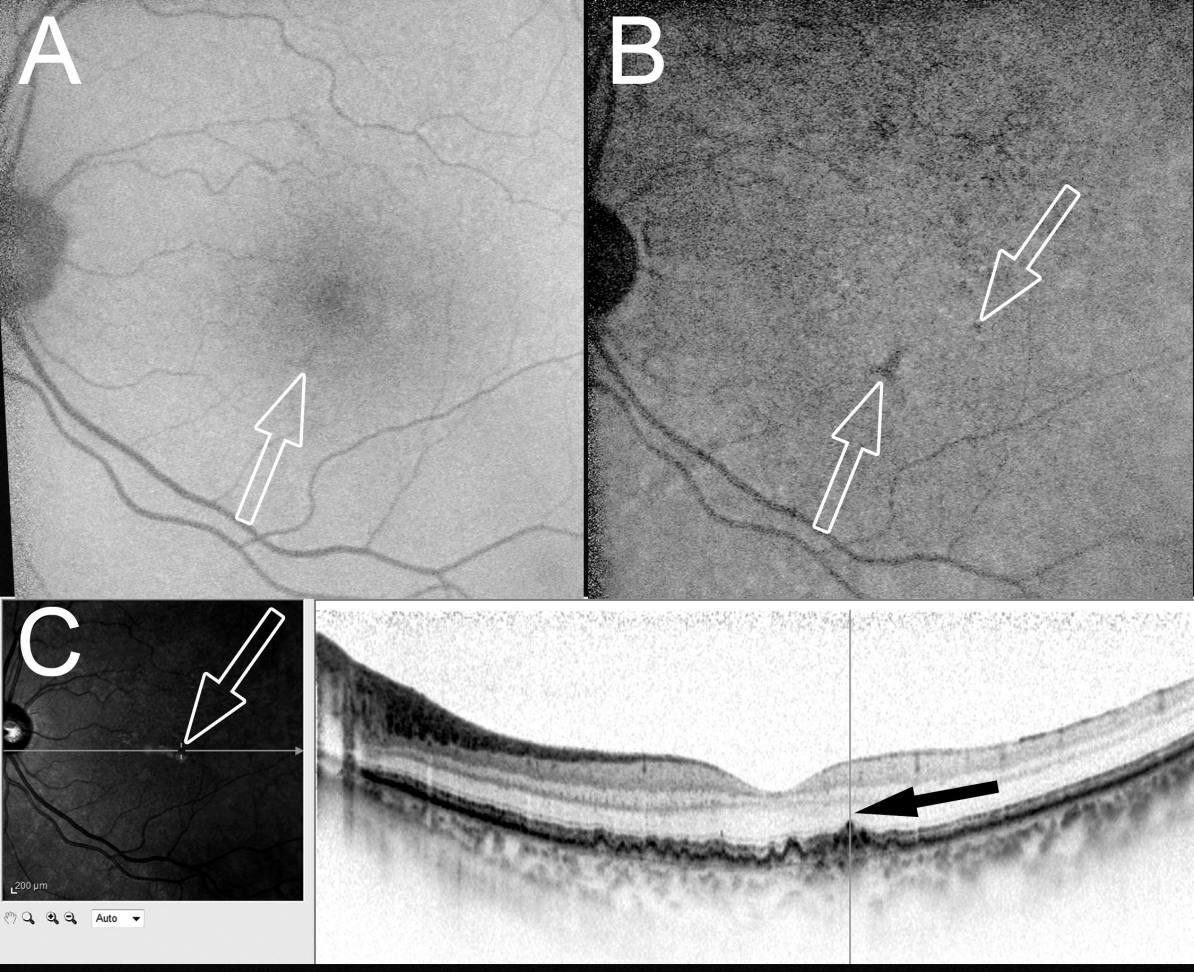
SW-AF and NIR-AF images obtained 24 months before the onset of RAP lesions. Twenty-four months before the initial treatment in the case in Fig 2. The arrows in A, B, and C correspond to the RAP lesions on the ICGA images at baseline. (A) An SW-AF image shows undetermined abnormalities (arrow). (B) An NIR-AF image clearly shows hypoautofluorescence (arrows). (C) A horizontal OCT image shows bulging of the RPE line and thinning of the outer nuclear layer (ONL) (black arrow).

The mean follow-up period in all 76 patients was 39.2 ± 29.4 (range, 0.1–131.8) months. Among 76 patients, 21 (21%) eyes developed RAP during the follow-up period. Eighteen of the 21 eyes were examined by both SW-AF and NIR-AF imaging before the onset of RAP lesions (Table 3, Figs 3 and 4). Among the 18 eyes, SW-AF imaging showed abnormalities corresponding to the RAP lesions on ICGA at the initial treatment in 8 (44.4%) eyes: 3 (16.7%) eyes had hyperautofluorescence and 5 (27.8%) eyes had hypoautofluorescence (Table 3). NIR-AF imaging showed abnormalities corresponding to the RAP lesions on ICGA at the initial treatment in 15 (83.3%) of the 18 eyes; all 15 eyes had hypoautofluorescence (Table 3, Figs 3B and 4B). Significant differences between SW-AF and NIR-AF imaging were seen with regard to the prevalence of abnormalities, the prevalence of hypoautofluorescence, and the prevalence of eyes with no abnormalities (Fisher’s exact test: p = 0.035, p = 0.0020, and p = 0.035, respectively) (Table 3). The raw data for the mean follow-up period and the cases in which RAP developed in the fellow eye during the follow-up period are presented in S3 Table.

**Table 3:** Abnormalities before the onset of RAP lesions (18 eyes).

	SW-AF	NIR-AF	p Value*
Abnormalities, eyes (%)	8 (44.4%)	15 (83.3%)	0.035
Hyperautofluorescence; eyes (%)	3 (16.7%)	0 (0%)	0.23
Hypoautofluorescence; eyes (%)	5 (27.8%)	15 (83.3%)	0.0020
None, eyes (%)	10 (55.6%)	3 (16.7%)	0.035

*Fisher's exact test.

RAP, retinal angiomatous proliferation; SW-AF, short-wavelength autofluorescence; NIR-AF, near-infrared fundus autofluorescence.

### OCT findings

Spectral-domain OCT, which was performed for 15 eyes with hypoautofluorescence on NIR-AF imaging before the onset of RAP lesions showed both bulging of the RPE line and thinning of the outer nuclear layer (ONL) in all 15 (100%) eyes (Figs 3 and 4).

## Discussion

The current study reported for the first time that SW-AF imaging showed abnormalities in 27% of eyes and NIR-AF imaging showed abnormalities in 87% of eyes in patients with treatment-naïve RAP. Hypoautofluorescence on NIR-AF imaging was the most prevalent abnormality in the current study, and is important for establishing an accurate diagnosis of RAP or anticipating the imminent onset of RAP lesions.

SW-AF and NIR-AF imaging are useful for obtaining high-resolution images of the eye, both noninvasively and rapidly, in patients with AMD. The presence of macular atrophy detected by SW-AF imaging has been reported to have one of the strongest correlations with poor visual outcomes in AMD patients treated with ranibizumab [30]. SW-AF imaging has become a common and important examination for patients with AMD. However, no previous studies have described the detailed abnormalities of patients with RAP using NIR-AF and SW-AF imaging. The results of the present study could be very useful for diagnosing and managing these patients.

Intraretinal neovascularization outside of the foveal avascular zone was detected by OCT in 7 eyes with RAP [31]. SW-AF imaging shows decreased fluorescence at the macula due to blockage caused by macular pigment [2]. However, because of the higher optical density of RPE melanin, NIR-AF imaging shows fluorescence more clearly in comparison to SW-AF imaging [21]. This reduced effect of macular pigment might be responsible for the high sensitivity of NIR-AF imaging in the detection of abnormalities in patients with RAP. The current study showed a marked difference in the ability of SW-AF and NIR-AF imaging to detect abnormalities in patients with RAP. The current results showed that hypoautofluorescence on SW-AF imaging may indicate dysfunction or damage of the RPE. Hypoautofluorescence in the NIR-AF is associated with melanin loss. The detection of this abnormality by either modality may be helpful for the noninvasive diagnosis of patients with early-stage RAP.

In the current study of 76 patients, 21% of eyes developed RAP during the follow-up period. Among the 18 eyes that underwent examinations with both modalities before the onset of RAP lesions, abnormalities corresponding to the RAP lesions on ICGA at the initial treatment were visualized by NIR-AF imaging in 83.3% of eyes and by SW-AF imaging in 44.4% of eyes. The presence of hypoautofluorescence on NIR-AF imaging may be highly useful for predicting the imminent onset of RAP lesions. RAP requires the repeated administration of anti-VEGF therapy during long-term follow-up, which may result in a high prevalence of RPE atrophy. RAP is well known to be associated with a high risk of the onset of RAP lesions in the fellow eye [20]. Thus, the current study of the characteristic findings of hypoautofluorescence on NIR-AF imaging may be helpful for managing patients with early-stage RAP, which may improve or stabilize their VA during long-term follow-up.

Among the 15 eyes with hypoautofluorescence on NIR-AF imaging before the onset of RAP lesions, SD-OCT showed both bulging of the RPE line and thinning of the ONL before the onset of RAP lesions in all 15 eyes. From these results, we hypothesized that, in early-stage RAP, dysfunction of the photoreceptor–RPE complex and melanin deficiency may result from intense macular stress, during which time several cytokines, including VEGF, could be simultaneously released, ultimately leading to intraretinal neovascularization or sub-RPE neovascularization. Hasegawa et al. [32] reported the characteristics of spontaneous retinal neovascularization in NRV2 mice and concluded that the multiple areas of retinal depigmentation that were found, developed vascular leakage, which is strongly associated with the development of early-stage human RAP. The presence of hypo-melanin obtained in the current study using NIR-AF imaging may be strongly associated with depigmentation, which may be involved in the development of early-stage RAP.

Recently, Su et al. [33] found intraretinal hyperreflective foci (precursor lesion) in 14 of 18 RAP eyes (77.8%) with pre-onset OCT images that were suggestive of an early type 3 lesion. In the current study, hypoautofluorescence on NIR-AF imaging before the onset of RAP lesions could be detected clearly and easily in 15 of 18 RAP eyes (83.3%). Further studies are needed to reevaluate the correlation between the precursor lesions on OCT images and the autofluorescence results found in the current study.

The present study was associated with some limitations. The study population was relatively small and all patients were Japanese. Furthermore, the present study was retrospective in nature and was performed in a single center. Long-term prospective, randomized studies with larger cohorts are needed to determine the characteristics of fundus autofluorescence in patients with RAP.

In conclusion, the current study showed, for the first time, the characteristics of fundus autofluorescence in eyes with RAP. The abnormalities identified by SW-AF and NIR-AF imaging may be important and useful for predicting the imminent onset of RAP lesions. The mechanism of early-stage RAP may be highly correlated with the presence of hypo-melanin.

## Supporting information

S1 Table Raw data for the patient characteristics. (DOCX)

S2 Table Raw data for the SW-AF and NIR-AF abnormalities. (DOCX)

S3 Table Raw data for the mean follow-up period and the development of RAP in the fellow eye during follow-up. (DOCX)
