# Supplementary material for: Fundus autofluorescence of retinal angiomatous proliferation
Source: PLoS One. 2020 Dec 9;15(12):e0243458. doi: 10.1371/journal.pone.0243458 (PMC7725377; doi:10.1371/journal.pone.0243458)
Supplement: S1 Table — (DOCX) [file pone.0243458.s001.docx]

S1 Table: Raw data of Patient Characteristics

| Patient's No. | Side | baseline VA log | GLD | stage of RAP | The number of RAP lesions | PED |
| --- | --- | --- | --- | --- | --- | --- |
| 1 | OD | 1.10 | 3806 | stage2+PED | 1 | yes |
| 1 | OS | 1.05 | 1502 | stage2+PED | 1 | yes |
| 2 | OD | 0.00 | 602 | stage2 | 1 | no |
| 2 | OS | 1.00 | 6248 | stage3 | 1 | yes |
| 3 | OD | 0.30 | 3801 | stage2+PED | 1 | yes |
| 4 | OS | 0.52 | 2160 | stage2+PED | 1 | yes |
| 5 | OD | 0.52 | 1475 | stage2 | 1 | no |
| 6 | OS | 0.52 | 3493 | stage2+PED | 1 | yes |
| 7 | OD | 0.82 | 4160 | stage2+PED | 1 | yes |
| 7 | OS | 0.52 | 803 | stage2 | 1 | no |
| 8 | OD | 0.40 | 975 | stage2 | 1 | no |
| 8 | OS | 0.05 | 2000 | stage2 | 1 | no |
| 9 | OS | 0.30 | 2393 | stage2 | 1 | no |
| 10 | OS | 0.40 | 817 | stage1 | 1 | no |
| 11 | OD | 0.00 | 1415 | stage2+PED | 1 | yes |
| 11 | OS | 1.70 | 4912 | stage3 | 1 | yes |
| 12 | OD | -0.08 | 679 | stage2 | 2 | no |
| 12 | OS | 1.10 | 3033 | stage2+PED | 1 | yes |
| 13 | OS | 0.52 | 2875 | stage2+PED | 1 | yes |
| 14 | OS | 0.70 | 4360 | stage2+PED | 1 | yes |
| 15 | OD | 0.70 | 1449 | stage2 | 1 | no |
| 16 | OD | 1.22 | 1271 | stage2 | 1 | no |
| 16 | OS | 0.70 | 2733 | stage2 | 1 | no |
| 17 | OD | 1.30 | 4500 | stage2 | 1 | no |
| 17 | OS | 0.52 | 5600 | stage2+PED | 1 | yes |
| 18 | OD | 0.00 | 1225 | stage2 | 1 | no |
| 18 | OS | 0.70 | 3326 | stage2 | 1 | no |
| 19 | OD | 0.30 | 548 | stage2 | 1 | no |
| 20 | OS | 1.00 | 1900 | stage2+PED | 1 | yes |
| 21 | OS | 0.30 | 2890 | stage1 | 1 | no |
| 22 | OD | 0.30 | 2875 | stage2 | 1 | no |
| 23 | OS | 0.15 | 2950 | stage2+PED | 1 | yes |
| 24 | OD | 1.15 | 5590 | stage3 | 1 | yes |
| 24 | OS | -0.18 | 1051 | stage2 | 1 | no |
| 25 | OS | 1.05 | 6613 | stage2+PED | 2 | yes |
| 26 | OD | 1.22 | 3413 | stage2+PED | 1 | yes |
| 27 | OD | 1.15 | 5569 | stage3 | 2 | yes |
| 27 | OS | 0.82 | 5565 | stage2+PED | 1 | yes |
| 28 | OS | 0.40 | 4700 | stage2+PED | 1 | yes |
| 29 | OD | 0.30 | 1521 | stage1 | 1 | no |
| 30 | OS | 0.40 | 690 | stage2 | 1 | no |
| 31 | OD | 0.70 | 5466 | stage2+PED | 1 | yes |
| 32 | OD | 1.22 | 5532 | stage3 | 1 | yes |
| 32 | OS | 0.15 | 2368 | stage2+PED | 1 | yes |
| 33 | OS | 1.22 | 1058 | stage2 | 1 | no |
| 34 | OD | 0.00 | 1287 | stage2 | 1 | no |
| 35 | OS | 0.40 | 2370 | stage2 | 1 | no |
| 36 | OS | 1.00 | 3036 | stage2 | 1 | no |
| 37 | OD | 1.15 | 4331 | stage2+PED | 1 | yes |
| 38 | OD | 1.00 | 3800 | stage3 | 1 | yes |
| 38 | OS | 0.22 | 460 | stage2 | 1 | no |
| 39 | OD | 0.52 | 2110 | stage2 | 1 | no |
| 40 | OD | 1.15 | 4604 | stage2+PED | 1 | yes |
| 40 | OS | 0.40 | 1380 | stage1 | 1 | no |
| 41 | OS | 0.00 | 415 | stage2 | 1 | no |
| 42 | OD | 0.40 | 2615 | stage2+PED | 1 | yes |
| 42 | OS | 0.52 | 2995 | stage2+PED | 1 | yes |
| 43 | OS | 0.10 | 2000 | stage2 | 1 | no |
| 44 | OD | 0.40 | 5673 | stage2 | 1 | no |
| 45 | OS | 1.00 | 6815 | stage2+PED | 1 | yes |
| 46 | OS | 0.15 | 1068 | stage2 | 3 | no |
| 47 | OD | 1.00 | 2892 | stage3 | 1 | yes |
| 48 | OD | 1.00 | 4800 | stage2+PED | 1 | yes |
| 48 | OS | 1.05 | 4100 | stage2+PED | 1 | yes |
| 49 | OS | 1.10 | 5500 | stage2+PED | 1 | yes |
| 50 | OS | 0.52 | 3696 | stage2+PED | 1 | yes |
| 51 | OS | 0.40 | 2776 | stage2 | 1 | no |
| 52 | OD | 0.22 | 2571 | stage2+PED | 1 | yes |
| 52 | OS | 0.70 | 2284 | stage2+PED | 1 | yes |
| 53 | OD | 0.40 | 1900 | stage2 | 1 | no |
| 54 | OS | 0.70 | 2233 | stage2+PED | 1 | yes |
| 55 | OD | 0.82 | 2378 | stage2+PED | 1 | yes |
| 56 | OD | 0.30 | 2350 | stage2 | 1 | no |
| 57 | OD | 0.40 | 4178 | stage2+PED | 1 | yes |
| 57 | OS | 1.05 | 3648 | stage2+PED | 1 | yes |
| 58 | OD | 0.22 | 1285 | stage2 | 1 | no |
| 59 | OD | 0.52 | 2743 | stage2+PED | 2 | yes |
| 60 | OS | 0.30 | 2569 | stage2 | 1 | no |
| 61 | OS | 0.70 | 1101 | stage2 | 1 | no |
| 62 | OS | 0.70 | 4037 | stage3 | 1 | yes |
| 63 | OS | 1.05 | 2465 | stage2+PED | 1 | yes |
| 64 | OD | -0.08 | 1418 | stage2 | 1 | no |
| 64 | OS | 1.00 | 3150 | stage2+PED | 2 | yes |
| 65 | OS | 0.92 | 3832 | stage2+PED | 1 | yes |
| 66 | OD | 1.00 | 3603 | stage2+PED | 1 | yes |
| 66 | OS | 1.00 | 1451 | stage2+PED | 1 | yes |
| 67 | OS | 0.22 | 3000 | stage2 | 1 | no |
| 68 | OD | 1.10 | 4565 | stage3 | 1 | yes |
| 68 | OS | 0.52 | 4863 | stage2+PED | 1 | yes |
| 69 | OS | 0.30 | 2276 | stage2 | 1 | no |
| 70 | OD | 0.05 | 2367 | stage1 | 1 | no |
| 70 | OS | 0.70 | 1359 | stage2+PED | 1 | yes |
| 71 | OD | 0.82 | 1517 | stage2 | 1 | no |
| 71 | OS | 1.05 | 3733 | stage2 | 1 | no |
| 72 | OD | 0.52 | 605 | stage2 | 1 | no |
| 73 | OD | 0.70 | 3569 | stage3 | 2 | yes |
| 74 | OS | 0.82 | 7569 | stage2+PED | 1 | yes |
| 75 | OD | 0.52 | 2398 | stage2+PED | 1 | yes |
| 76 | OD | 0.52 | 2361 | stage2 | 1 | no |
| 76 | OS | 0.00 | 686 | stage1 | 1 | no |

OD: oculus dexter, OS: oculus sinister, VA: visual acuity; GLD: greatest linear dimension, RAP: retinal angiomatous proliferation, PED: pigment epithelial detachment.
