# Supplementary material for: Fundus autofluorescence of retinal angiomatous proliferation
Source: PLoS One. 2020 Dec 9;15(12):e0243458. doi: 10.1371/journal.pone.0243458 (PMC7725377; doi:10.1371/journal.pone.0243458)
Supplement: S2 Table — (DOCX) [file pone.0243458.s002.docx]

S2 Table: Raw data of Abnormalities on SW-AF and NIR-AF

| Patient's No. | SW-AF | NIR-AF |
| --- | --- | --- |
| 1 | none | hypo |
| 1 | none | hypo |
| 2 | hypo | hypo |
| 2 | none | none |
| 3 | hypo | hypo |
| 4 | block | hypo |
| 5 | block | hypo |
| 6 | hypo | hypo |
| 7 | none | none |
| 7 | block | hypo |
| 8 | hypo | none |
| 8 | none | hypo |
| 9 | hyper | hypo |
| 10 | none | hypo |
| 11 | block | hypo |
| 11 | none | hypo |
| 12 | hypo and block | hypo and hypo |
| 12 | none | none |
| 13 | hypo | hypo |
| 14 | none | none |
| 15 | block | none |
| 16 | none | hypo |
| 16 | hypo | hypo |
| 17 | none | none |
| 17 | none | hypo |
| 18 | none | hypo |
| 18 | none | hyper |
| 19 | hypo | hypo |
| 20 | hypo | hypo |
| 21 | hypo | hypo |
| 22 | none | hypo |
| 23 | hypo | hypo |
| 24 | none | block |
| 24 | none | hypo |
| 25 | none | hyper and hypo |
| 26 | block | block |
| 27 | none | hyper and hypo |
| 27 | none | hypo |
| 28 | block | hypo |
| 29 | none | hypo |
| 30 | block | hypo |
| 31 | none | hypo |
| 32 | none | none |
| 32 | none | hypo |
| 33 | hypo | hypo |
| 34 | none | hypo |
| 35 | none | hypo |
| 36 | none | hypo |
| 37 | none | hypo |
| 38 | none | hypo |
| 38 | hypo | hypo |
| 39 | hypo | hypo |
| 40 | none | hypo |
| 40 | none | hypo |
| 41 | none | hypo |
| 42 | hypo | hypo |
| 42 | none | hypo |
| 43 | none | hypo |
| 44 | none | hypo |
| 45 | block | block |
| 46 | none | hypo, hypo and none |
| 47 | none | hypo |
| 48 | none | hypo |
| 48 | none | hypo |
| 49 | hypo | hypo |
| 50 | none | hypo |
| 51 | hypo | hypo |
| 52 | none | hypo |
| 52 | none | hypo |
| 53 | none | hypo |
| 54 | hypo | hypo |
| 55 | block | hypo |
| 56 | none | hypo |
| 57 | none | hypo |
| 57 | none | hypo |
| 58 | none | hypo |
| 59 | block | hypo and block |
| 60 | block | block |
| 61 | none | hypo |
| 62 | hypo | hypo |
| 63 | hypo | hypo |
| 64 | none | hypo |
| 64 | block | hypo and block |
| 65 | none | hypo |
| 66 | none | hypo |
| 66 | hypo | hypo |
| 67 | none | none |
| 68 | none | hypo |
| 68 | none | hypo |
| 69 | none | hypo |
| 70 | hypo | hypo |
| 70 | none | hypo |
| 71 | hypo | hypo |
| 71 | hypo | hypo |
| 72 | none | hypo |
| 73 | hyper and hypo | hypo and hypo |
| 74 | none | hypo |
| 75 | none | hypo |
| 76 | hypo | hypo |
| 76 | none | hypo |

SW-AF: short-wavelength autofluorescence, NIR-AF: near-infrared autofluorescence.
