# Supplementary material for: Fundus autofluorescence of retinal angiomatous proliferation
Source: PLoS One. 2020 Dec 9;15(12):e0243458. doi: 10.1371/journal.pone.0243458 (PMC7725377; doi:10.1371/journal.pone.0243458)
Supplement: S3 Table — (DOCX) [file pone.0243458.s003.docx]

S3 Table: Raw data of mean follow-up period and presence of developed RAP in the unaffected eye during the follow-up

| Patient's No. | Mean follow-up period (months) | Developed RAP in the unaffected eye during the follow-up period |
| --- | --- | --- |
| 1 | 28.6 | no |
| 1 | 28.6 | no |
| 2 | 99.7 | yes |
| 2 | 99.7 | no |
| 3 | 46.0 | no |
| 4 | 0.1 | no |
| 5 | 20.9 | no |
| 6 | 15.0 | no |
| 7 | 14.2 | no |
| 7 | 14.2 | yes |
| 8 | 60.3 | no |
| 8 | 60.3 | yes |
| 9 | 46.4 | yes |
| 10 | 9.0 | no |
| 11 | 51.4 | yes |
| 11 | 51.4 | no |
| 12 | 57.9 | no |
| 12 | 57.9 | no |
| 13 | 75.1 | yes |
| 14 | 55.5 | yes |
| 15 | 69.5 | no |
| 16 | 13.1 | no |
| 16 | 13.1 | no |
| 17 | 81.8 | no |
| 17 | 81.8 | no |
| 18 | 60.7 | yes |
| 18 | 60.7 | no |
| 19 | 56.4 | no |
| 20 | 5.1 | no |
| 21 | 39.0 | no |
| 22 | 20.1 | no |
| 23 | 21.0 | yes |
| 24 | 17.4 | no |
| 24 | 17.4 | yes |
| 25 | 47.2 | no |
| 26 | 32.2 | no |
| 27 | 63.3 | no |
| 27 | 63.3 | no |
| 28 | 10.3 | no |
| 29 | 17.0 | no |
| 30 | 42.7 | no |
| 31 | 19.6 | no |
| 32 | 12.1 | no |
| 32 | 12.1 | no |
| 33 | 23.1 | no |
| 34 | 4.0 | no |
| 35 | 47.4 | no |
| 36 | 22.1 | no |
| 37 | 94.1 | no |
| 38 | 28.5 | no |
| 38 | 28.5 | yes |
| 39 | 70.1 | yes |
| 40 | 47.8 | no |
| 40 | 47.8 | no |
| 41 | 39.7 | no |
| 42 | 49.1 | yes |
| 42 | 49.1 | no |
| 43 | 105.8 | no |
| 44 | 14.5 | no |
| 45 | 11.2 | no |
| 46 | 27.5 | yes |
| 47 | 98.3 | yes |
| 48 | 10.7 | no |
| 48 | 10.7 | no |
| 49 | 62.5 | no |
| 50 | 9.0 | no |
| 51 | 25.4 | no |
| 52 | 11.1 | no |
| 52 | 11.1 | no |
| 53 | 131.8 | no |
| 54 | 32.2 | no |
| 55 | 4.0 | no |
| 56 | 35.2 | no |
| 57 | 1.8 | no |
| 57 | 1.8 | no |
| 58 | 86.3 | yes |
| 59 | 21.6 | no |
| 60 | 73.7 | yes |
| 61 | 12.3 | no |
| 62 | 7.5 | no |
| 63 | 13.1 | no |
| 64 | 49.2 | yes |
| 64 | 49.2 | no |
| 65 | 4.7 | no |
| 66 | 39.2 | no |
| 66 | 39.2 | no |
| 67 | 102.0 | no |
| 68 | 3.5 | no |
| 68 | 3.5 | no |
| 69 | 11.8 | no |
| 70 | 24.3 | yes |
| 70 | 24.3 | no |
| 71 | 78.8 | no |
| 71 | 78.8 | yes |
| 72 | 25.0 | no |
| 73 | 86.6 | yes |
| 74 | 60.4 | no |
| 75 | 12.1 | no |
| 76 | 43.5 | no |
| 76 | 43.5 | no |

RAP: retinal angiomatous proliferation.
